# THAM reduces CO_2_-associated increase in pulmonary vascular resistance – an experimental study in lung-injured piglets

**DOI:** 10.1186/s13054-015-1040-4

**Published:** 2015-09-17

**Authors:** Staffan Höstman, João Batista Borges, Fernando Suarez-Sipmann, Kerstin M. Ahlgren, Joakim Engström, Göran Hedenstierna, Anders Larsson

**Affiliations:** Hedenstierna Laboratory, Uppsala University, Uppsala, Sweden; Department of Surgical Sciences, Uppsala University Hospital, Entrance 70, 75185 Uppsala, Sweden; Department of Medical Sciences, Uppsala University, Uppsala, Sweden; Cardio-Pulmonary Department, Pulmonary Division, Heart Institute (Incor), University of São Paulo, São Paulo, Brazil

## Abstract

**Introduction:**

Low tidal volume (V_T_) ventilation is recommended in patients with acute respiratory distress syndrome (ARDS). This may increase arterial carbon dioxide tension (PaCO_2_), decrease pH, and augment pulmonary vascular resistance (PVR). We hypothesized that Tris(hydroxymethyl)aminomethane (THAM), a pure proton acceptor, would dampen these effects, preventing the increase in PVR.

**Methods:**

A one-hit injury ARDS model was established by repeated lung lavages in 18 piglets. After ventilation with V_T_ of 6 ml/kg to maintain normocapnia, V_T_ was reduced to 3 ml/kg to induce hypercapnia. Six animals received THAM for 1 h, six for 3 h, and six serving as controls received no THAM. In all, the experiment continued for 6 h. The THAM dosage was calculated to normalize pH and exhibit a lasting effect. Gas exchange, pulmonary, and systemic hemodynamics were tracked. Inflammatory markers were obtained at the end of the experiment.

**Results:**

In the controls, the decrease in V_T_ from 6 to 3 ml/kg increased PaCO_2_ from 6.0±0.5 to 13.8±1.5 kPa and lowered pH from 7.40±0.01 to 7.12±0.06, whereas base excess (BE) remained stable at 2.7±2.3 mEq/L to 3.4±3.2 mEq/L. In the THAM groups, PaCO_2_ decreased and pH increased above 7.4 during the infusions. After discontinuing the infusions, PaCO_2_ increased above the corresponding level of the controls (15.2±1.7 kPa and 22.6±3.3 kPa for 1-h and 3-h THAM infusions, respectively). Despite a marked increase in BE (13.8±3.5 and 31.2±2.2 for 1-h and 3-h THAM infusions, respectively), pH became similar to the corresponding levels of the controls. PVR was lower in the THAM groups (at 6 h, 329±77 dyn∙s/m^5^ and 255±43 dyn∙s/m^5^ in the 1-h and 3-h groups, respectively, compared with 450±141 dyn∙s/m^5^ in the controls), as were pulmonary arterial pressures.

**Conclusions:**

The pH in the THAM groups was similar to pH in the controls at 6 h, despite a marked increase in BE. This was due to an increase in PaCO_2_ after stopping the THAM infusion, possibly by intracellular release of CO_2_. Pulmonary arterial pressure and PVR were lower in the THAM-treated animals, indicating that THAM may be an option to reduce PVR in acute hypercapnia.

## Introduction

Low tidal volume (V_T_) ventilation has been shown to reduce ventilator-induced lung injury (VILI) and to improve survival in patients with acute respiratory distress syndrome (ARDS) [1]. In addition, it seems to reduce the risk of lung complications after surgery in which patients are under general anesthesia [2]. Therefore, low V_T_ ventilation is recommended for ventilating patients with ARDS and has also gained support in anesthesia.

It has been suggested that ventilation with V_T_ even less than 6 ml/kg would further reduce the risk of VILI in ARDS [3]. However, very low V_T_ ventilation may increase arterial carbon dioxide tension (PaCO_2_) substantially. Although hypercapnic acidosis has been shown to have both negative and positive effects on immune function, it has unequivocal and clinically relevant negative effects on the pulmonary circulation, increasing pulmonary vascular resistance (PVR) [4–7]. This, in combination with high positive end-expiratory pressure (PEEP) levels, may induce acute right heart failure [8, 9].

Different methods to reduce PaCO_2_ during low V_T_ ventilation have been suggested, such as reduction of apparatus dead space by a tracheal double-lumen tube, tracheal gas insufflation, expiratory flushing of the dead space by a tracheal catheter, or prolonging the end-inspiratory pause [10–13]. One of the most common methods is to increase minute ventilation by increasing respiratory rate (RR). However, this sometimes results in an unwanted buildup of auto-PEEP. Furthermore, it might be that the increased RR in itself, owing to increased energy transfer to the lungs, induces VILI. Indeed, very high RR combined with very low V_T_ using high-frequency oscillation has not been shown to be beneficial and might even increase mortality in ARDS [14, 15].

Other methods are extracorporeal CO_2_ removal using an arteriovenous or venovenous approach with a low blood flow through a small membrane oxygenator/CO_2_ remover. However, this method has not yet been shown to improve clinical outcome [16]. Thus, in many situations with low V_T_ ventilation, hypercapnic acidosis is unavoidable.

Because the acidosis caused by the increased PaCO_2_ may be the main reason for the side effects of “permissive hypercapnia,” it has been speculated whether treatment with sodium bicarbonate (NaHCO_3_) would be useful [1, 17, 18]. In fact, in order for NaHCO3 to work as a buffer, it has to generate CO_2_, which in turn has to be removed via the lungs increasing ventilatory demands [19]. Because the problem with low V_T_ ventilation is that the CO_2_ excretion via the lungs is impaired, NaHCO_3_ is theoretically not a good choice for buffering in these conditions. However, Tris(hydroxymethyl)aminomethane (THAM), or trometamol, which is a pure proton acceptor that exerts its buffer effects without generating CO_2_, could be a more logical choice in cases of excessive CO_2_ accumulation [20]. In addition, there are experimental data indicating that THAM may attenuate VILI [21]. THAM has already been used with success in cases with severe asthma and as an adjunct in ARDS [5, 20, 22]. However, in all published studies so far, THAM has been used at a modest dose, for a short period of time, and not with the aim of full pH correction. Using a porcine model, we have previously shown that THAM could be used to correct pH for at least 3 h during total apnea and that PVR was not severely increased during this period [23]. The aims of the present study were to explore how THAM, administered during two limited periods of very low V_T_ ventilation, would affect, first, pH and PVR and, second, lung inflammatory parameters in a porcine lung lavage model. We hypothesized that, during hypercapnia, THAM infusion would keep pH normal by increasing the metabolic base component, would prevent increase of PVR, and would not increase lung injury.

## Methods

The study was approved by the Animal Research Ethics Committee at Uppsala University, and the National Institutes of Health guidelines for animal research were followed. The study was performed at the Hedenstierna Laboratory, Uppsala University, Uppsala, Sweden.

### Anesthesia, ventilation, instrumentation, and monitoring

Eighteen pigs (23.0–30.5 kg body weight) were premedicated with 6 mg kg^−1^ tiletamine and zolazepam (Zoletil Forte; Boehringer Ingelheim Vetmedica, Ingelheim, Germany) and 2.2 mg kg^−1^ intramuscular xylazine (Rompun; BayerDVM, Shawnee Mission, KS, USA). After 5–10 minutes, the animal was placed supine on a table and a tracheotomy was performed by inserting an 8-mm I.D. endotracheal tube (Mallinckrodt Medical, Dublin, Ireland). Ventilation was started using a volume-controlled mode on a SERVO-i ventilator (Maquet Critical Care, Solna, Sweden) with V_T_ 8 ml kg^−1^, inspiratory/expiratory ratio (I:E) 1:1, fraction of inspired oxygen (FiO_2_) 0.5, RR 25 cycles/min, and PEEP 5 cmH_2_O. Just before the tracheostomy, a bolus of fentanyl 10–20 μg kg^−1^ was given intravenously. Anesthesia was then maintained with ketamine 30 mg kg^−1^ h^−1^, midazolam 0.1 mg kg^−1^ h^−1^, and fentanyl 4 μg kg^−1^ h^−1^. After checking that anesthesia was sufficient to prevent responses to painful stimulation between the front toes, muscle relaxation was added by a continuous infusion of pancuronium 0.3 mg kg^−1^ h^−1^.

During the first hour, 10 ml kg^−1^ h^−1^ Ringer’s acetate was infused intravenously, after which the infusion rate was lowered to 5 ml kg^−1^ h^−1^ intravenously. After open dissection of the neck vessels, an arterial catheter was inserted into the right carotid artery for blood sampling and blood pressure monitoring, and a central venous catheter was inserted via the right external jugular vein. In addition, a pulmonary arterial catheter (Criti Cath No7; Ohmeda, Singapore) for measurement of cardiac output (CO) and pulmonary arterial pressure was introduced via the right external jugular vein, and its correct position was verified by pressure monitoring. CO was obtained as the mean of three values measured by thermodilution after injection of 10 ml of ice-cold saline into the central venous catheter (Siemens SC 9000XL; Dräger, Lübeck, Germany). A bladder catheter was inserted suprapubically to measure hourly urine production. Electrocardiographic monitoring was started, and peripheral capillary oxygen saturation was monitored at the base of the tail by pulse oximetry (Siemens SC 9000XL). Next, a lung recruitment maneuver was performed with I:E 1:1, RR 6 breaths/min, pressure control, inspiratory pressure 40 cmH_2_O, and PEEP 20 cmH_2_O for 60 seconds. If the animal was considered hemodynamically unstable (mean arterial blood pressure [MAP] <50 mmHg) at this point, 50-ml boluses of hydroxyethyl starch (Hesra, Baxter Medical, Kista, Sweden) were given until a MAP of at least 50 mmHg was reached. All recruitment maneuvers were performed in the manner detailed above.

### Estimation of required amounts of buffer to equilibrate protons

Using data from a pilot study and previous experiments, we targeted a pH of 7.35 and a PaCO_2_ of 15 kPa at the 6-h endpoint. The pH target of 7.35 was chosen because, as seen in the study by Weber et al. [5], already at pH of approximately 7.2 there was a rise in mean pulmonary arterial pressure (MPAP). Inserting these values into the Henderson-Hasselbalch equation and solving for HCO_3_^−^ = 10^(7.2−6.1)^∙0.23 × 15 = 43 mEq/L, we could find the mEq/L amount of buffer needed as the difference between this value and normal bicarbonate (HCO_3_^−^), which was assumed to be 20 mEq/L. The buffering capacity needed for 1 h of infusion was then estimated as weight in kilograms × 23 × 0.4 = 9.2 × weight. The recommended dosing for THAM (Addex-THAM; Fresenius Kabi, Uppsala, Sweden) is normally calculated with a coefficient of 0.3, not 0.4, but in a pilot study a 1h infusion with coefficient 0.3 was found to be an inadequate dose; therefore, the coefficient was raised.

### Experimental protocol

#### Preparations

After the instrumentation, a lung recruitment maneuver was performed to homogenize lung volume history, FiO_2_ was set to 1.0, and the animal was allowed to stabilize for at least 15 minutes before baseline blood gas values were recorded; that is, arterial and venous blood was sampled for measurement of oxygen tension (PaO_2_), PaCO_2_, mixed venous oxygen saturation, hemoglobin, lactate, sodium, chloride, potassium, calcium, glucose content, pH, and base excess (BE) (ABL800 FLEX; Radiometer, Copenhagen, Denmark), and oxygen hemoglobin saturation (OSM3; Radiometer). The following baseline hemodynamic parameters were measured: MAP, MPAP, central venous pressure (CVP), pulmonary capillary wedge pressure, and CO. Brody’s formula [24] for body surface area of pigs was used for the cardiac index (CI) calculations. PEEP was set to 0 cmH_2_O, and an airway pressure-volume (PV) loop was obtained by slow insufflation to 40 cmH_2_O followed by slow exsufflation via an occluder [25], and functional residual capacity was obtained (FRC) using a sulfur hexafluoride washin/washout method [26, 27].

After baseline measurements, lung injury was induced by repeated lung lavages with 30 ml/kg of 37–39 °C normal saline (eight in total). Blood gases were sampled again, and MAP, MPAP, and CVP, as well as FRC and compliance of the respiratory system as obtained from the maximum slope of the expiratory PV loop, were recorded [25].

Next, the tracheal tube was replaced with a double lumen endotracheal tube with both distal openings in the trachea [10]. The inspiratory tubing was connected to one of the endotracheal tube lumens and the expiratory tubing was attached to the other lumen, separating inspiration from expiration. Thus, the apparatus dead space was eliminated. A second lung recruitment maneuver was performed, and the ventilator was set to PEEP 10 cmH_2_O, V_T_ 6 ml/kg, and I:E 1:2, and the RR was adjusted to target a pH between 7.38 and 7.42 in two successive blood samples obtained within a 15-minute time window.

#### Main experiment

After confirming that pH was normal by arterial and venous blood gas sampling, hemodynamic measurements (time point 0) were registered. Hypoventilation was initiated by reducing V_T_ to 3 ml/kg, and THAM infusion was started at the same time through a central venous line, in the two buffering groups. Arterial and mixed venous blood gases were obtained at 5, 10, 15, 30, 60, 90, and 120 minutes and then every hour up to 6 h from time point 0. In addition, the hemodynamic variables were registered at similar time points, except at 5 and 10 minutes.

After 6 h of low V_T_ ventilation, further data were collected by drawing venous samples for cytokine analyses, measuring FRC and compliance and performing a lavage of the basal right lung with 30 ml of normal saline. Thereafter the left lung was removed and dissected to obtain tissue samples from the ventral, medial, and dorsal parts at the hilar level. The tissue samples were frozen in liquid nitrogen and stored at −80 °C. The animals were killed with an intravenous dose of potassium chloride under deep anesthesia.

### Cytokine analysis

Pieces weighing between 80 and 320 mg were homogenized in lysis buffer (15 mM Tris, pH 7.4, 150 mM NaCl, 1 mM CaCl_2_, 1 mM MgCl_2_, 0.5 % Triton X-100), with addition of protease inhibitor (Thermo Scientific, Waltham, MA, USA) using an IKA Ultra-Turrax T8 homogenizer (IKA-Werke, Staufen im Breisgau, Germany). Homogenates were centrifuged at 300×*g* at 4 °C for 10 minutes. Supernatants were kept at −80 °C until assay. Cytokine content was assessed using a DuoSet enzyme-linked immunosorbent assay (ELISA) system (R&D Systems, Minneapolis, MN, USA), for porcine tumor necrosis factor (TNF)-α, interleukin (IL)-1β/IL-1F2, and IL-6 according to the manufacturer’s instructions. The detection limits of the assays were 125 pg/ml for TNF-α, 62.5 pg/ml for IL-1β/IL-1F2, and 125 pg/ml for IL-6. Total protein content of the supernatant was measured using a Coomassie Plus Assay (Thermo Scientific) according to the manufacturer’s instructions. Cytokine content of tissue lysates were normalized against total protein content of the homogenate.

### Statistics

A power analysis (analysis of variance [ANOVA]) indicated that six animals in each group would be enough to detect a pH difference of 0.1 with a standard deviation of 0.05 with a *p* <0.05 and a power of 0.8, assuming a normal distribution.

If there was a clear resemblance with the exponential distribution, a log transformation was performed to improve normality. If the variables did not pass the Shapiro-Wilk normality test, Kruskal-Wallis one-way ANOVA was performed. Tukey’s honestly significant difference was assessed post hoc. The data are presented as mean and standard deviation. The statistical calculations were performed using R 3.1.1 [28] and SigmaPlot 11.0 (Systat Software, San Jose, CA, USA).

## Results

All values reported in groups of three are in the order controls (NT), 1-h infusion (1T), 3-h infusion (3T) of THAM. *p*-Values reported are with regard to NT unless otherwise specified.

### Blood gas and acid–base status

**Fig. 1:**
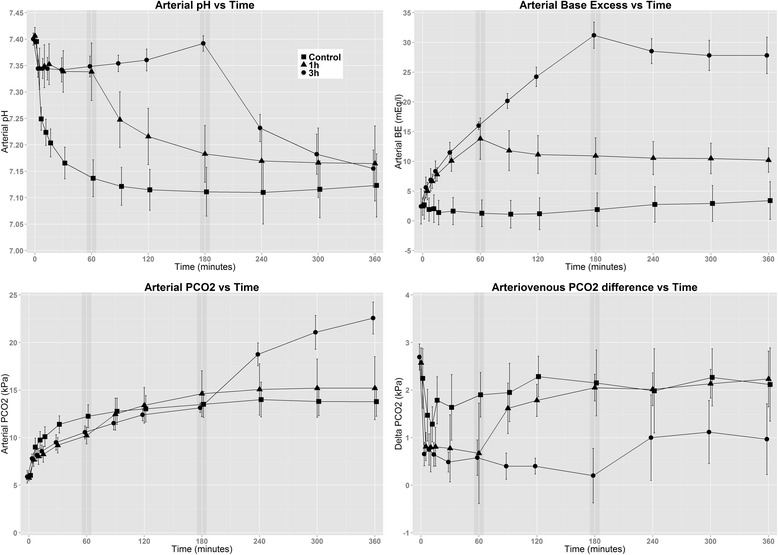
Effects of Tris(hydroxymethyl)aminomethane on arterial pH, base excess, arterial carbon dioxide tension (PCO_2_) and arteriovenous difference in PCO_2_. The graph shows the progression over time, with the *x*-axes representing time points. Note the offset *y*-axes. The end of infusion for the 1-h and 3-h groups are marked with a *darkened vertical bar*. Values are means, and the *error bars* represent standard deviations

Blood gas and acid–base data are reported in Table 1 and graphed in Fig. 1.

**Table 1:** Arterial acid–base values, arterial and venous oxygenation, oxygen consumption, and venous admixture

Parameter	Group	Baseline	Start	60 min	180 min	360 min
pH	NT	7.40 (0.04)	7.40 (0.01)	7.14 (0.03)	7.11 (0.05)	7.12 (0.06)
	1T	7.40 (0.05)	7.41 (0.02)	7.34 (0.05)^a^	7.18 (0.05)^a^	7.16 (0.07)
	3T	7.43 (0.02)	7.40 (0.01)	7.35 (0.02)^a^	7.39 (0.01)^a,b^	7.16 (0.03)
PaCO_2_ (kPa)	NT	6.6 (1.2)	6.0 (0.5)	12.3 (1.2)	13.5 (1.4)	13.8 (1.5)
	1T	6.0 (0.6)	5.8 (0.3)	10.2 (0.9)^a^	14.6 (2.4)	15.2 (3.3)
	3T	6.3 (0.7)	5.9 (0.7)	10.6 (0.7)^a^	13.1 (0.5)	22.6 (1.7)^a,b^
PaO_2_ (kPa)	NT	62.6 (12.7)	63.7 (7.4)	48.1 (12.0)	45.2 (12.3)	46.4 (11.8)
	1T	65.2 (4.8)	65.0 (8.6)	59.8 (6.3)	49.4 (9.0)	53.3 (5.0)
	3T	55.5 (15.5)	56.0 (4.6)	49.7 (4.1)	32.6 (7.6)§	30.0 (7.7)^a,b^
Base excess (mEq/ml)	NT	4.8 (2.2)	2.7 (2.3)	1.3 (2.2)	1.8 (2.8)	3.4 (3.2)
	1T	3.0 (3.2)	2.4 (1.5)	13.8 (3.5)^a^	10.9 (3.1)^a^	10.2 (2.1)^a^
	3T	6.2 (2.1)	2.4 (3.0)	16.0 (0.6)^a^	31.2 (2.2)^a,b^	27.8 (3.1)^a,b^
HCO_3_^−^ (mmol/L)	NT	28.5 (1.8)	27.2 (2.5)	29.7 (2.2)	30.8 (2.5)	32.3 (2.8)
	1T	27.0 (2.8)	26.7 (1.5)	40.1 (3.2)^a^	39.3 (3.2)^a^	38.8 (2.5)^a^
	3T	29.8 (1.9)	26.8 (3.1)	42.3 (0.9)^a^	58.6 (2.2)^a,b^	57.2 (2.8)^a,b^
SaO_2_ (%)	NT	98 (0.3)	98 (0.2)	97 (0.4)	97 (0.6)	97 (0.6)
	1T	98 (0.2)	98 (0.3)	98 (0.3)^a^	98 (0.2)	97 (0.2)
	3T	98 (0.8)	98 (0.1)^c^	98 (0.2)^a^	98 (0.3)	97 (0.8)
SvO_2_ (%)	NT	71 (11)	53 (11)	63 (6.1)	67 (5.8)	67 (11)
	1T	68 (4.6)	43 (5.2)	54 (10)	65 (6.5)	66 (10)
	3T	70 (7.6)	47 (11)^c^	61 (7.9)	69 (4.6)	74 (2.1)
VO_2_ (ml/min)	NT	133 (32)	124 (22)	118 (23)	120 (9)	121 (21)
	1T	128 (28)	142 (18)	131 (20)	139 (14)^c^	140 (26)
	3T	133 (30)^c^	123 (18)^c^	117 (7)	127 (7)	134 (13)
Q_s_/Q_t_ (fraction)	NT	0.15 (0.03)	0.11 (0.04)	0.17 (0.04)	0.20 (0.07)	0.20 (0.08)
	1T	0.13 (0.03)	0.09 (0.02)	0.11 (0.03)^a^	0.17 (0.05)^c^	0.15 (0.04)
	3T	0.16 (0.02)^c^	0.12 (0.02)^c^	0.15 (0.03)^b^	0.24 (0.04)	0.26 (0.06)^b^
Lactate (mmol/L)	NT	1.1 (0.26)	1.3 (0.23)	0.7 (0.08)	0.6 (0.14)	0.6 (0.14)
	1T	1.6 (0.81)	1.4 (0.14)	1.1 (0.19)^a^	0.6 (0.14)	0.7 (0.19)
	3T	1.1 (0.32)	1.2 (0.15)	0.9 (0.31)	0.9 (0.23)^a^	1.3 (0.59)^a,b^
ΔPaCO_2_ (kPa)	NT	1.5 (0.6)	2.2 (0.6)	1.9 (0.5)	2.2 (0.7)	2.1 (0.8)
	1T	1.8 (0.4)	2.6 (0.3)	0.7 (1.1)^a^	2.1 (0.3)	2.2 (0.6)
	3T	1.5 (0.3)	2.7 (0.3)	0.6 (0.4)^a^	0.2 (0.6)^a,b^	1.0 (0.7)^a,b^

*Abbreviations: BE* arterial base excess, *HCO*_*3*_^*−*^ arterial bicarbonate concentration, *NT* control animals that did not receive THAM, *PaCO*_*2*_ arterial carbon dioxide tension, *ΔPaCO*_*2*_ arteriovenous difference in carbon dioxide tension, *PaO*_*2*_ arterial oxygen tension, *Q*_*s*_ */Q*_*t*_ venous admixture obtained at inspired oxygen fraction 1.0, *SvO*_*2*_ mixed venous oxygen saturation, *1T* group that received THAM infusion for 1 h, *3T* group that received THAM infusion for 3 h, *THAM* Tris(hydroxymethyl)aminomethane, *VO*_*2*_ body oxygen consumption

The time points refer to time after start of THAM infusion or corresponding time points in the controls (NT)

^a^Value is different from NT group

^b^Value is different from 1T group

^c^Only 5 subjects represented owing to technical error

As evident in Fig. 1, arterial pH was largely different between the groups during THAM infusion. These differences diminished over time, showing no differences between groups at 360 minutes. PaCO_2_ rose in all groups. At the end of infusions, both the 1T and 3T groups showed a second phase of PaCO_2_ increase, whereas NT changed very little after 120 minutes. There was a large difference in PaCO_2_ at 360 minutes (13.8±1.5 kPa vs 15.2±3.3 kPa, *p* =0.55; 22.6±1.7 kPa, *p* <0.001). The difference between mixed venous and arterial carbon dioxide tension was lower in both 1T and 3T groups starting from the 15-minute time point (1.8±0.5 kPa vs 0.8±0.4 kPa, *p* =0.001; 0.6±0.2 kPa, *p* <0.001). After the infusion, the 1T group rebounded to levels similar to that of NT. At 360 minutes, the values were 2.1±0.8 kPa vs 2.2±0.6 kPa (*p* =0.96) and 1.0±0.7 kPa (*p* =0.03). At 360 minutes, the BE values were 3.4±3.2 mEq/L vs 10.2±2.1 mEq/L (*p* =0.002) and 27.8±3.1 mEq/L (*p* <0.001). HCO_3_^−^ showed an increase similar to that of BE.

Arterial oxygen tension (PaO_2_) trended toward decrease and was lower at 360 minutes in the 3T group (*p* <0.001) compared with controls and the 1T group. The 3T group had a higher shunt fraction than the 1T group at 360 minutes (*p* =0.03).

### Hemodynamics

**Fig. 2:**
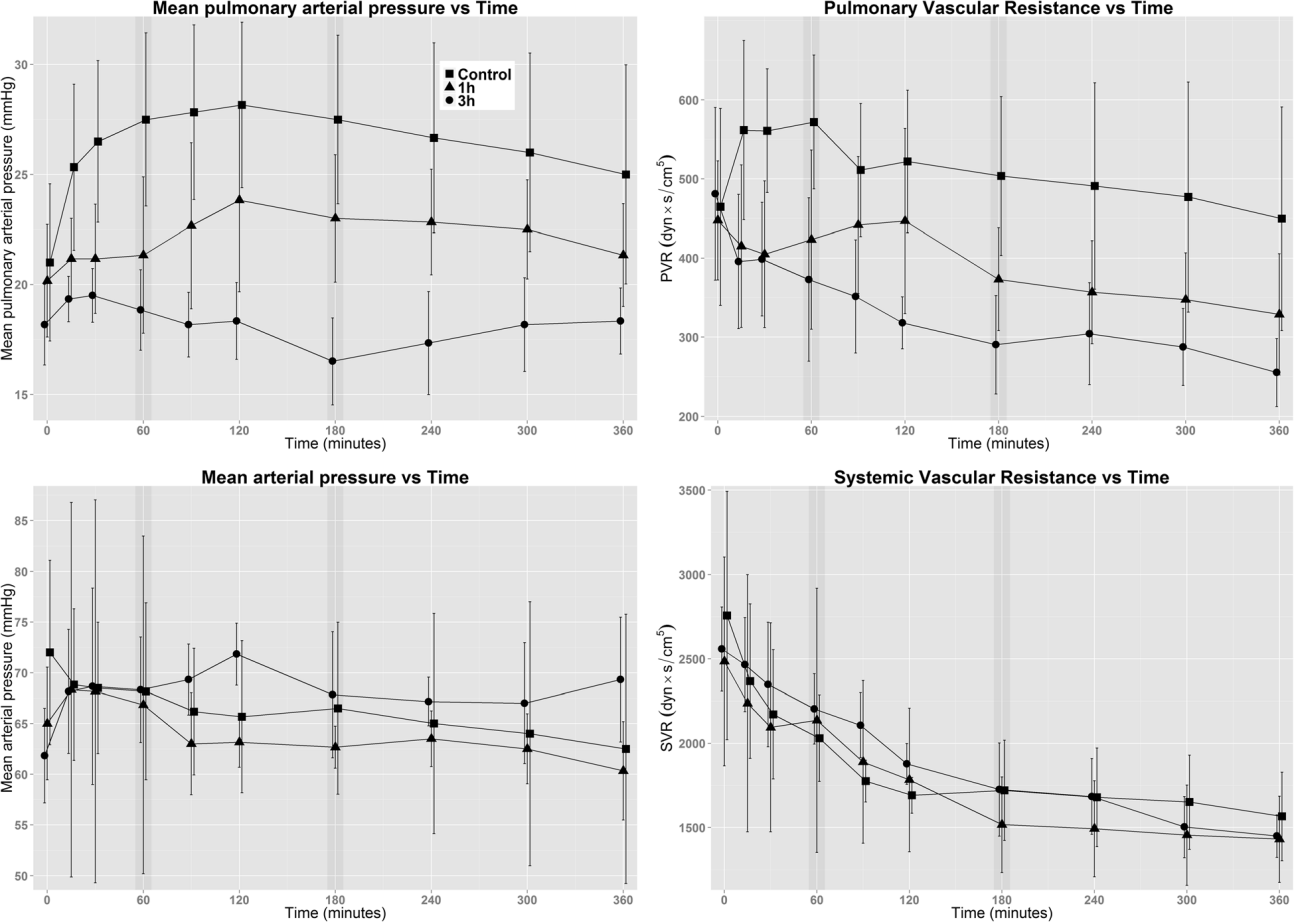
Effects of Tris(hydroxymethyl)aminomethane on hemodynamics. The graph shows the progression over time, with the *x*-axes representing time points. Note the offset *y*-axes. The end of infusion for the 1-h and 3-h groups is marked with a *darkened vertical bar*. Values are means, and the represent standard deviations. *SVR* systemic vascular resistance

Hemodynamic data are reported in Table 2 and graphed in Fig. 2.

**Table 2:** Hemodynamics

Parameter	Group	Baseline	Start	60 min	180 min	360 min
MPAP (mmHg)	NT	18 (3)	21 (4)	28 (4)	28 (4)	25 (5)
	1T	19 (2)	20 (3)	21 (4)^a^	23 (3)^a^	21 (2)
	3T	14 (2)^a,b^	18 (2)	19 (2)^a^	17 (2)^a,b^	18 (2)^a^
MAP (mmHg)	NT	83 (9)	72 (9)	68 (9)	67 (9)	63 (13)
	1T	95 (13)	65 (6)	67 (17)	63 (2)	60 (5)
	3T	80 (7)	62 (5)	68 (5)	68 (6)	69 (6)
CI (L/min/m^2^)	NT	3.6 (0.5)	2.4 (0.3)	2.9 (0.3)	3.4 (0.7)	3.5 (1.0)
	1T	3.4 (0.7)	2.4 (0.3)	2.8 (0.4)	3.8 (0.7)	3.8 (0.8)
	3T	4.2 (1.1)	2.4 (0.4)	3.1 (0.5)	4.2 (0.7)	5.0 (0.6)^a,b^
PVR (dyn∙s/m^5^)	NT	317 (92)	465 (125)	572 (85)	504 (101)	450 (141)
	1T	323 (79)	448 (75)	423 (113)^a^	373 (65)^a^	329 (77)
	3T	264 (69)	481 (109)	373 (103)^a^	290 (62)^a^	255 (43)^a^
SVR (dyn∙s/m^5^)	NT	2240 (515)	2757 (736)	2030 (257)	1720 (297)	1566 (262)
	1T	2942 (1201)	2485 (619)	2136 (782)	1517 (283)	1431 (255)
	3T	2147 (716)	2558 (250)	2204 (208)	1725 (276)	1449 (125)

*Abbreviations: CI* cardiac index, *MAP* mean systemic arterial pressure, *MPAP* mean pulmonary arterial pressure, *NT* control animals that did not receive THAM, *PVR* pulmonary vascular resistance, *SVR* systemic vascular resistance, *1T* group that received THAM infusion for 1 h, *3T* group that received THAM infusion for 3 h, *THAM* Tris(hydroxymethyl)aminomethane

The time points refer to time after start of THAM infusion or corresponding time points in the controls (NT)

Values are mean (standard deviation)

^a^Value is different from NT group

^b^Value is different from 1T group

The MPAP was lower in the 3T and 1T groups from the 30- and 60-minute time points, respectively, onward; at 360 minutes, however, only the 3T group was different from the control animals (25±5 mmHg vs 21±2 mmHg, *p* =0.17; 18±2 mmHg, *p* =0.008). PVR was similar to MPAP, and the 3T group was still different at 360 minutes (450±141 dyn∙s/m^5^ vs 329±77 dyn∙s/m^5^, *p* =0.11; 255±43 dyn∙s/m^5^, *p* =0.0081). CI exhibited a rising trend over time in all the groups, with 3T separating from the rest at 360 minutes (3.5±1.0 L/min/m^2^ vs 3.8±0.75 L/min/m^2^, *p* =0.42; 5.0±0.57 L/min/m^2^, *p* <0.001). MAP and systemic vascular resistance (SVR) did not differ between the groups at the examined time points, but SVR showed a decreasing trend over time in all three groups.

### Inflammatory markers

**Fig. 3:**
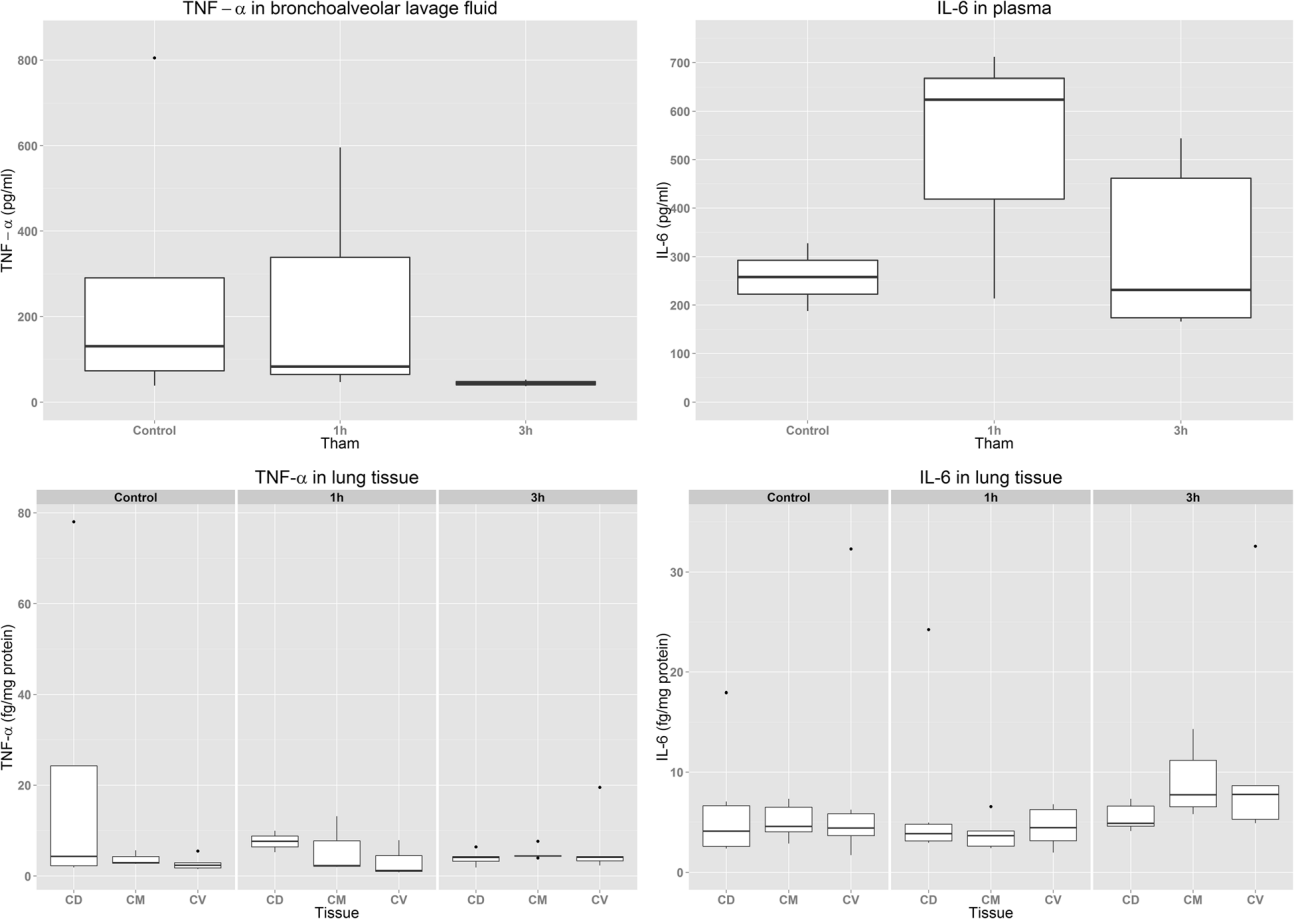
Standard box-and-whisker plots of the cytokine levels stratified by amount of Tris(hydroxymethyl)aminomethane (THAM) given. Note that the two *top panels* depict tumor necrosis factor (TNF)-α in bronchoalveolar lavage fluid and interleukin (IL)-6 in plasma. *CD* dorsal, *CM* medial, *CV* ventral

Inflammatory marker data are depicted in Fig. 3.

Because many values were at levels too low to be detected with the ELISA, they are not presented in a table. Most of the missing (i.e., below the detection limit) values were in the 1T and 3T groups, indicating that they might be generally lower than in the controls.

In the tissue samples, there was an effect on the IL-6 concentration (detected by two-way ANOVA) in the THAM strata, and post hoc analysis showed a *p*-value of 0.014 compared with controls, with the 3T group being higher (11.8±11.7 pg/mg_protein_) than the 1T group (4.5±2.0 pg/mg_protein_) and non-significant values found for the rest (*p* =0.086 for 3T vs NT and *p* =0.423 for 1T vs NT). Two-way ANOVA of the TNF-α and IL-1β concentrations was not done, owing to many missing values. Kruskal-Wallis one-way ANOVA on ranks was performed for every tissue strata, with the missing values set below the minimum measured values. No differences were detected.

### Lung mechanics

Data regarding lung mechanics are provided in Table 3.

**Table 3:** Lung mechanics

Parameter	Group	Baseline	Lung lavage	End
FRC (ml)	NT	386 (45)	224 (81)	278 (46)
	1T	435 (105)	231 (54)	287 (116)
	3T	341 (53)	158 (21)	202 (46)
Cexp (ml/cmH_2_O)	NT	47 (10)	37 (9)	47 (5)
	1T	51 (19)	39 (17)	49 (17)
	3T	51 (7)	41 (2)	50 (3)

*Abbreviations: Cexp* compliance of the respiratory system obtained as the maximal slope of a full expiratory pressure-volume curve, *FRC* functional residual capacity, *NT* control animals that did not receive Tris(hydroxymethyl)aminomethane (THAM), *1T* group that received THAM infusion for 1 h, *3T* group that received THAM infusion for 3 h

Lung lavage values obtained 30 minutes after lung lavage; end values obtained at the end of the experiment

Values are mean (standard deviation)

FRC and compliance of the respiratory system decreased with the lavages (*p* <0.001) but did not differ between the groups.

### Electrolytes

Electrolyte data are reported in Table 4.

**Table 4:** Electrolytes

Parameter	Group	Start	60 min	180 min	360 min
[Na^+^] (mmol/L)	NT	134 (1)	134 (1)	132 (2)	130 (2)
	1T	134 (2)	129 (2)^a^	134 (1)	135 (1)^a^
	3T	132 (2)^a,b^	125 (2)^a,b^	125 (2)^a,b^	138 (3)^a,b^
[K^+^] (mmol/L)	NT	4.3 (0.2)	4.6 (0.2)	5.2 (0.4)	5.5 (0.6)
	1T	4.5 (0.2)	5.1 (0.3)^a^	5.0 (0.3)	5.2 (0.4)
	3T	4.3 (0.3)	5.0 (0.3)	5.9 (0.3)^a,b^	4.8 (0.5)
[Ca^2+^] (mmol/L)	NT	1.43 (0.17)	1.51 (0.16)	1.35 (0.11)	1.29 (0.11)
	1T	1.43 (0.14)	1.33 (0.19)^a^	1.36 (0.14)	1.28 (0.13)
	3T	1.25 (0.06)^b^	1.21 (0.04)^a^	1.01 (0.06)^a,b^	1.09 (0.05)^a,b^
[Cl^−^] (mmol/L)	NT	101 (2)	99 (1)	98 (2)	93 (3)
	1T	101 (1)	98 (1)	97 (3)	93 (2)
	3T	104 (3)	99 (1)	96 (2)	94 (2)

*Abbreviations: [Ca* ^*2+*^ *]* arterial calcium concentration, *[Cl* ^*−*^ *]* arterial chloride concentration, *[K* ^*+*^ *]* arterial potassium concentration, *[Na* ^*+*^ *]* arterial sodium concentration, *NT* control animals that did not receive THAM, *1T* group that received THAM infusion for 1 h, *3T* group that received THAM infusion for 3 h, *THAM* Tris(hydroxymethyl)aminomethane

The time points refer to time after start of THAM infusion or corresponding time points in the controls (NT)

Values are mean (standard deviation)

^a^Value is different from NT group

^b^Value is different from 1T group

Arterial sodium concentration fell during the THAM infusion in both the 1T and 3T groups, and arterial potassium concentration increased.

## Discussion

This study shows the following results in a porcine lung lavage model:

1. Permissive hypercapnia obtained by reducing the effective V_T_ from 6 ml/kg to 3 ml/kg decreased pH with a slow metabolic compensation during the study period and increased PVR, but it did not deteriorate CO or have other severe effects. Thus, these findings indicate that hypercapnia in this model did not compromise right heart function.
2. Buffering with THAM infused for 1 or 3 h intravenously immediately normalized pH both by reducing the increase in PaCO_2_ and by a fast increase in BE. It was also associated with a decreased PVR.
3. After the end of the continuous infusion of THAM, there was a rebound increase in PaCO_2_, and, in spite of a continued high BE, pH decreased to levels similar to those of the controls at the end of the experimental period.
4. Notwithstanding the decrease in pH and marked increase in PaCO_2_, PVR remained low.
5. The inflammatory response in the lungs as well as the lung volumes and lung mechanics were not markedly different between the THAM groups and the controls.

We previously showed, in a porcine apneic model, that a continuous THAM infusion maintained pH at physiologically acceptable levels for at least 3 h. PVR was only slightly increased, despite a marked increase in PaCO_2_ to a maximum of 30 kPa [23]. In that study, where we investigated whether THAM could totally replace ventilation of the lungs for a longer period, we did not discontinue the THAM infusion during the experiment. In the present study, where we studied whether THAM could be an adjunct to ventilation, pH decreased after the THAM infusion was stopped. This was due to a rebound increase in PaCO_2_ that was severe after the prolonged THAM infusion. Despite this fact, PVR did not increase, MPAP remained low, and CO increased.

Most studies of the pulmonary circulatory effects of hypercapnia have been performed in models with hypoxia-induced pulmonary vasoconstriction (HPV) in either intact animals or isolated lung preparations where vasoconstriction was induced by lowering the inspired oxygenation concentration [29–33]. This is in contrast to our lavage model, where we kept PaO_2_ high by FiO_2_ 1.0 and a lung recruitment maneuver followed by PEEP 10 cmH_2_O. The other important differences are that, in most of the other previous studies, hypercapnia was induced by administering CO_2_ in the breathing gas and manipulations of the metabolic component were done by infusion of NaHCO_3_ or HCl [17, 34, 35]. In the majority of these studies, researchers found that both metabolic and hypercapnic acidosis increased PVR and that alkalosis, independent of whether it was metabolic or hypercapnic, reduced or normalized pulmonary vascular tone. Thus, the pH change in itself was interpreted to be the main mechanism of this effect [29, 36, 37]. Our results challenge this interpretation because PVR in the THAM groups was low despite development of a moderate to severe respiratory acidosis after the THAM infusion was stopped. Instead, BE and HCO_3_^−^ were maintained at high levels, indicating that the metabolic component might be more important than pH alone in the regulation of pulmonary vascular tone. That THAM reduces increases in MPAP caused by hypercapnia has previously been demonstrated by Weber et al. [5]. However, they did not find any significant change in PVR induced by THAM, probably owing to simultaneous administration of cardiovascular active agents. Furthermore, they did not report any remaining metabolic effect induced by THAM after the infusion was ended.

In contrast to the pulmonary circulation, we did not find any relation between arterial BE or pH and vascular resistance in the systemic circulation. However, the reduction in SVR mirrored the increase in PaCO_2_, suggesting that, in the systemic circulation, PaCO_2_ per se influences the vascular tone more than BE or pH does, confirming results in previous studies [34, 38]. Although the underlying mechanism of the effect on the vasculature by respiratory or metabolic acid–base changes have not been fully elucidated, it has been suggested that intracellular pH changes may regulate the voltage-gated potassium channels and that this effect is different between the pulmonary and systemic vessels [39]. Thus, a decrease in the intracellular pH dilates systemic vessels and constricts pulmonary vessels. In addition, on the basis of our present study, we cannot exclude a direct pharmacological effect of THAM on the vascular smooth muscles. In agreement with clinical studies, the decrease in SVR caused by THAM was associated with an increase in CO and mixed venous oxygen saturation [5].

The significant increase in PaCO_2_ after THAM infusion was completed in the THAM-treated animals compared with the controls was unexpected and, to our knowledge, has not been described before. The two THAM dosages were calculated to increase BE to balance the increased CO_2_ to keep pH normal. We assumed that PaCO_2_ would level out at a new high steady state similar to the controls when the CO_2_ excretion via the lungs would again equalize the CO_2_ production in the tissues. In the control animals, this new, higher steady state (at PaCO_2_ around 15 kPa) occurred after about 120 minutes. However, in the THAM-treated animals, PaCO_2_ showed a two-phase course. During the infusion, the increase in PaCO_2_ followed, but, as expected, below the increase in PaCO_2_ of the controls because THAM reduces the CO_2_ content in the blood by catching protons, moving the Henderson-Hasselbalch equation to the right. However, after the infusion was stopped, PaCO_2_ increased and leveled off at a higher level than in the controls. In the animals treated with THAM for 3 h, the PaCO_2_ increase was substantial, from a mean of 15 to 23 kPa with no clear steady state at the end of the experiment.

Three theoretical mechanisms might explain the increased PaCO_2_ after the THAM infusion was stopped:

1. *Reduced alveolar ventilation*: However, the tidal volumes were kept constant throughout the study, and we do not have any indications in any of the groups that physiological dead space increased or that a marked increase in pulmonary shunt occurred after the end of the infusion.
2. *Increased metabolism*: However, body temperature was kept constant, and calculated oxygen consumption was unchanged; therefore, it is not plausible that CO_2_ production increased.
3. *Increased CO*_*2*_*accumulation in the tissues due to reduced pulmonary excretion during the THAM infusion*: This is indicated by the significantly lower pulmonary mixed venous-arterial PaCO_2_ gradient value during the THAM infusion in the 3-h THAM group. Thus, THAM might have sequestered CO_2_ that thereafter accumulated in the tissues. Because the tension of CO_2_ cannot be higher in the tissues than in the blood, the CO_2_ must have been stored as HCO_3_^−^, probably in the intracellular compartment. However, THAM penetrates slowly into the intracellular compartment, and during the first hour most of the protonated THAM was probably excreted via the kidneys [19]. This can explain why we found only a minor (non-significant) increase in PaCO_2_ after the infusion in the 1-h THAM infusion group, in contrast to the marked increase in the 3-h infusion group. Thus, one could speculate that the intracellular concentration of CO_2_, stored as HCO_3_^−^ together with protonated THAM, might have been very high at the end of the infusion in the 3-h group. When the infusion was stopped, the intracellular HCO_3_^−^ was, via the effect of carbonic anhydrase, transformed into CO_2_, which penetrated the cell membranes easily and increased PaCO_2_. Nevertheless, this unexpected effect of THAM needs further exploration.

THAM can have important side effects, such as hypoglycemia, hyperkalemia, and hypotension [19]. In this study, although potassium levels increased, glucose levels were normal and blood pressure was maintained.

We did not find any major differences in inflammatory markers between the groups. Only IL-6 showed a significant increase in lung tissue, whereas we could not find any difference in TNF-α or IL-1β. The time with acidosis was significantly less in the THAM groups, which indicates that, when very low V_T_ ventilation in addition to adequate PEEP level are used, the potential specific anti-inflammatory effect of hypercapnic acidosis is minor and that THAM is probably safe to administer in this regard. This notion is further strengthened by the fact that lung mechanics and FRC recovered to a similar degree after lavage in all groups. Our findings are in accord with those reported by Caples et al., who found that buffering with THAM ameliorated ventilation-induced lung cell injuries in isolated rat lungs [21].

The possible clinical implication of our study is that THAM might be useful in cases of high PVR complicating permissive hypercapnia by reducing the pulmonary vascular tone. However, this has to be done very cautiously, particularly because the mechanism of the rebound effect of PaCO_2_ is unknown. Thus, the dose and infusion time should be restricted, and PaCO_2_ and potassium levels should be carefully monitored, as should PaO_2_, which might decrease owing to inhibition of HPV.

This study has the following inherent limitations of all animal models, and the results cannot be assumed to be fully valid in patients:

1. Although pigs have physiology very similar to that of as humans, their immunologic responses, as well as their response to THAM, might be different from those of humans.
2. The effect on the pulmonary vasculature might be more prominent in pigs because pigs have a strong HPV [40].
3. We used an open lung approach, the animals were never hypoxemic, and inhibition of HPV may deteriorate oxygenation importantly in ARDS [37].
4. We used FiO_2_ 1.0, which might have augmented the pulmonary vasodilatory effect associated with THAM.
5. Although THAM was infused in a large vein, its high osmolality may have caused endothelial damage. However, the osmolality is less than the commonly used concentration of hypertonic saline and amino acid solutions used for parenteral nutrition.
6. Possible toxic side effects were not addressed.
7. The number of animals used, as well as the observation period, was limited.

## Conclusions

This experimental study of permissive hypercapnia in a porcine lung lavage model shows that intravenous infusion of THAM increased BE and bicarbonate concentration, normalized pH, and decreased PaCO_2_ during the infusion. After a prolonged infusion, however, pH decreased to values similar to those in controls owing to a rebound PaCO_2_ increase. Despite a similar low pH and a higher PaCO_2_ compared with controls, the PVR remained low in the THAM group. No major signs of augmentation of lung injury by THAM were found. These findings suggest that CO_2_ removal from the lungs is hampered during THAM infusion and that the metabolic component (i.e., BE, bicarbonate) has an important influence on the pulmonary vascular tone, indicating the potential use of THAM in situations with hypercapnia-induced pulmonary hypertension and increased PVR.

## Key messages

- Increased pulmonary vascular resistance caused by respiratory acidosis was countered with THAM.
- After a high-dose infusion of THAM, PaCO_2_ rebounded to a higher-than-expected level.
- THAM did not importantly affect the inflammatory response.

## Abbreviations

1T: 1-h infusion of THAM
3T: 3-h infusion of THAM
ANOVA: Analysis of variance
ARDS: Acute respiratory distress syndrome
BE: Base excess
CD: Dorsal
Cexp: compliance of the respiratory system
CI: Cardiac index
CM: Medial
CO: Cardiac output
CV: Ventral
CVP: Central venous pressure
ELISA: Enzyme-linked immunosorbent assay
FiO_2_: Fraction of inspired oxygen
FRC: Functional residual capacity
HCO_3_^−^: Bicarbonate
HPV: Hypoxic pulmonary vasoconstriction
I:E: Inspiratory/expiratory ratio
IL: Interleukin
MAP: Mean arterial blood pressure
MPAP: Mean pulmonary arterial pressure
NaHCO_3_: Sodium bicarbonate
ns: Non-significant
NT: Controls (i.e., no THAM infusion)
PaCO_2_: Arterial carbon dioxide tension
PaO_2_: Arterial oxygen tension
PEEP: Positive end-expiratory pressure
PV: Pressure-volume
PVR: Pulmonary vascular resistance
Q_s_/Q_t_: Shunt fraction
RR: Respiratory rate
SvO_2_: Mixed venous oxygen saturation
SVR: Systemic vascular resistance
THAM: Tris(hydroxymethyl)aminomethane
TNF-α: Tumor necrosis factor α
VILI: Ventilator-induced lung injury
VO_2_: body oxygen consumption
V_T_: Tidal volume
ΔPaCO_2_: Arteriovenous difference in carbon dioxide tension
